# Morpho-Physiological and Biochemical Responses of Field Pea Genotypes under Terminal Heat Stress

**DOI:** 10.3390/plants12020256

**Published:** 2023-01-05

**Authors:** Vijay Sharma, Chandra Mohan Singh, Vishal Chugh, Kamaluddin, Pawan Kumar Prajapati, Anuj Mishra, Prashant Kaushik, Parmdeep Singh Dhanda, Alpa Yadav, Satyendra

**Affiliations:** 1 Department of Genetics and Plant Breeding, College of Agriculture, Banda University of Agriculture and Technology, Banda 210001, India; 2 Department of Basic and Social Sciences, College of Horticulture, Banda University of Agriculture and Technology, Banda 210001, India; 3 Independent Researcher, 46022 Valencia, Spain; 4 Department of Biochemistry, Punjab Agricultural University, Ferozepur Road, Ludhiana 141027, India; 5 Department of Chemistry, Ranchi University, Ranchi 834001, India

**Keywords:** biochemical characterization, chlorophyll, ROS, heat stress, field pea

## Abstract

Field pea is one of the important short-duration cool season pulse crops which contributes significantly towards food and nutritional security. Two heat-susceptible (HS) and two heat-tolerant (HT) genotypes were selected from the previous study for further characterization. A significant variation was observed for morpho-physiological traits studied. Principal component analysis explained that first two principal components, i.e., PC1 and PC2 showed 76.5% of the total variance in optimal condition, whereas 91.2% of the total variance was covered by the first two PCs in heat stress environment. The seed yield per plant determined significant and positive association with superoxide dismutase and number of seeds per pod under optimal conditions, whereas under heat stress condition, it was positively associated with number of effective pods per plant, biological yield per plant, proline, pod length, number of seeds per pod, superoxide dismutase, and peroxidase. The significant reduction was noticed in the susceptible genotypes, whereas tolerant genotypes showed stable and non-significant reduction in chlorophyll content. Further, minimum cell damage and higher hydrogen peroxide production was noticed in the susceptible genotypes. In addition, the biochemical characterization of HS and HT genotypes revealed that the higher expression of peroxidase, superoxide dismutase, and catalase modulates the tolerant responses in HT genotypes. These genotypes were further used in developing heat-tolerant field pea genotypes.

## 1. Introduction

Field pea (*Pisum sativum* L. *subsp. sativum var. arvense* (L.) Poir.) is an autogamous grain legume grown in a wide range of climatic conditions varying from semiarid to temperate globally [1]. The pea seeds are a rich source of protein, ranging from 21 to 25% content, with an excess of lysine and tryptophan and other amino acids indicating the high nutritional value of this crop [2,3]. It also has some extent of cysteine and methionine [4]. It is widely used as food and feed. In India, field pea occupies about 0.64 million hectares of total area and produces 0.88 million tonnes with a productivity of 1375 kg/ha in 2020–21 [5]. Considering the importance of this crop, it is observed that several biotic as well as abiotic stresses affect its production. In current climate change situation where global temperatures are rising day by day, the severe yield penalty has been noticed. However, it is a cool-season crop whose reproductive phase clash with the exposure to high temperature. It affects the anthesis, pod development, and pod filling index, which leads to the formation of shriveled seeds [6,7]. Therefore, heat stress at reproductive phase is the main threat to pea production globally [8,9].

The high temperature also affects the plants on several levels. It alters crop phenology, limits biomass production, shortens the reproductive phase and reduces flowering and fruiting, reduces seed number, and causes excessive yield losses [6,10,11]. Heat stress also alters normal growth and cell metabolism. Many physiological processes in plant cells, such as dysfunction of cytoplasmic enzymes, proteins degradation, and injury of membrane stability, are affected by heat stress [12]. In addition, heat stress affects plant water balance [13], decreases photosynthetic capacity [14], and lowers metabolic activities [15]. It also leads to the alteration of hormones, excess reactive oxygen species (ROS) accumulation [16], higher rate of production of ethylene [17], reduction in pollen viability and pollen germination [18,19,20,21]. These lead to starvation, reduced fertility, production of toxic chemicals, and growth inhibition [22]. In plants, oxidative stress neutralizes through the induction of detoxifying enzymes and antioxidants such as ascorbate peroxidase, peroxidase, superoxide dismutase, and catalase [23]. ROS are free radical as well as non-radical molecules that are vital parts of signaling pathway network. They are key controllers of cellular responses of plants to abiotic stress [24].

The documentation of heat sensitivity in pea plants has been noticed since the 1950s [25,26]. Makasheva [27] reported a 60–80% loss of seed yield in pea due to high temperatures. Similarly, a loss of 600 kg per hectare is recorded in pea crops due to a temperature increase of 1 °C each time during flowering [28]. Temperatures above 28 °C reduce seed yield and duration from blooming to maturity of field pea in dry soil environments in western Canada [7]. Keeping the facts under consideration, present study aimed to characterize four contrasting pea genotypes for morphological traits, physiological traits, and biochemical parameters.

## 2. Results

### 2.1. Trait Variations

Analysis of variance (Table 1) revealed that between the four genotypes, all the traits varied significantly at *p* ≤ 0.05, except the total number of pods per plant and number of effective pods per plant. Likewise, treatments were also found significant for all the biochemical traits except ascorbate peroxidase and malondialdehyde at *p* ≤ 0.05. The significant effect of genotype–treatment interactions was observed for all the characters at *p* ≤ 0.05 except superoxide dismutase, ascorbate peroxidase, days to 50% flowering, pod length, seed number per pod, chlorophyll-a and -b, and carotenoid.

### 2.2. Effects of Heat Stress on Plant Growth and Yield Component Traits

The performance of four genotypes of field pea for all the growth as well as yield component traits under non-stress (optimal or normal sown) as well as heat stress (suboptimal or late sown) conditions are summarized in Table 2. Each genotype showed a differential response under heat stress conditions. Among all the genotypes, DF ranged from 72.5 days (P-1541-16 and IM 9102) to 77.5 days (EC 341743) and 66.5 days (P-1541-16) to 72.5 days (P-1384-3) in optimal and heat stress conditions, respectively. All the genotypes had a differential reduction in DF in heat stress. The observed range in the mean value of DM in optimal varied from 115.0 days (P-1541-16) to 122.5 days (IM 9102), whereas in heat stress, it varied from 98.0 days (P-1541-16) to 105.0 days (IM 9102). The highest reduction in the mean value of DM was observed for susceptible genotypes as compared to tolerant genotypes. In the case of all genotypes, the lowest PH recorded for genotype P-1541-16 was 115.0 cm in control and 98.0 cm in stress, while greatest value of PH was found for genotype EC 341743 in both optimal and stress conditions. Significant variation was observed for TNP under optimal and heat stress. Higher TNP observed in genotypes P-1384-3 and EC 341743 in optimal and heat stress, respectively. In the case of NEP, the performance of genotypes varied from 29.4 (EC 341743) to 31.1 (P-1384-3 and P-1541-16) in optimal condition, whereas in heat stress, NEP varied from 12.3 (P-1541-16) to 20.2 (P-1384-3). Susceptible genotypes showed a greater reduction in stress conditions in NEP. In case of PL, heat stress revealed smaller pod lengths in all genotypes ranging from 3.5 cm to 5.2 cm, where tolerant genotypes had longer pod lengths than susceptible ones. The highest value for seed index for evaluated genotypes in optimal and heat stress was 15.5 g (IM 9102) and 14.0 g (P-1541-16), respectively. Tolerant genotypes showed a lower reduction in heat stress conditions in the seed index. Regarding the NSP, BY and SY, genotype P-1384-3 displayed the highest mean value in optimal as well as in heat stress while genotype P-1541-16 had the lowest values in both conditions. Among all the evaluated genotypes, heat stress conditions imposed a significant reduction on genotypes, namely P-1541-16 and IM 9102.

### 2.3. Effects of Heat Stress on Physiological Traits

**Figure 1:**
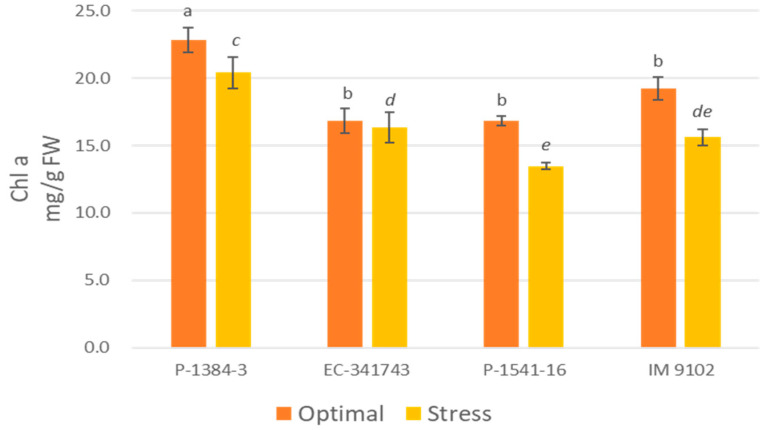
Chlorophyll-a content of four field pea genotypes under optimal and heat stress. Columns with different letters indicate significant difference at *p ≤* 0.05 (Fisher LSD test).

**Figure 2:**
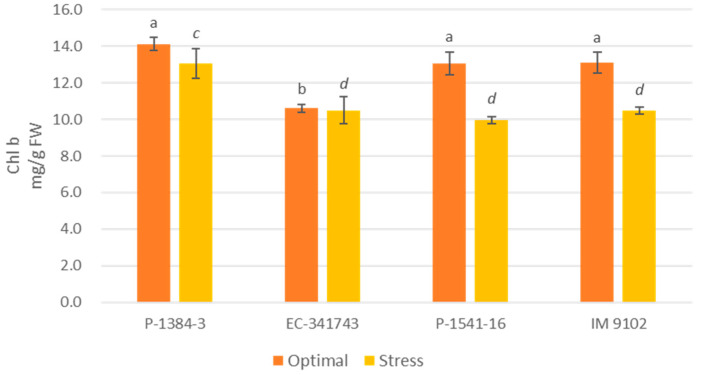
Chlorophyll-b content of four field pea genotypes under optimal and heat stress. Columns with different letters indicate significant difference at *p ≤* 0.05 (Fisher LSD test).

**Figure 3:**
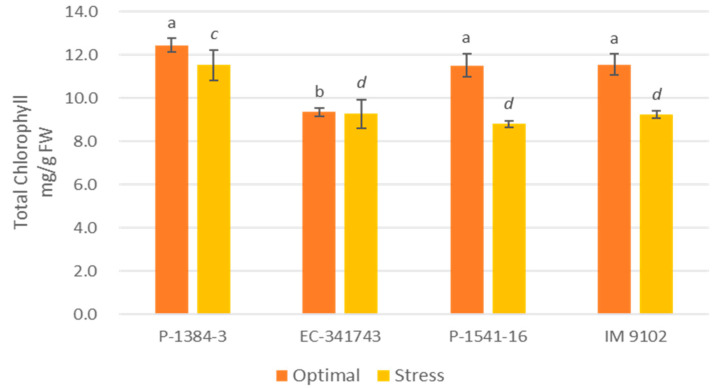
Total chlorophyll content of four field pea genotypes under optimal and heat stress. Columns with different letters indicate significant difference at *p ≤* 0.05 (Fisher LSD test).

**Figure 4:**
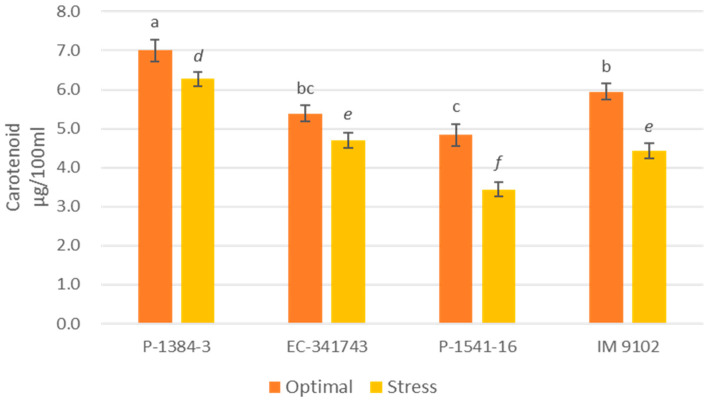
Carotenoid content of four field pea genotypes under optimal and heat stress. Columns with different letters indicate significant difference at *p ≤* 0.05 (Fisher LSD test).

**Figure 5:**
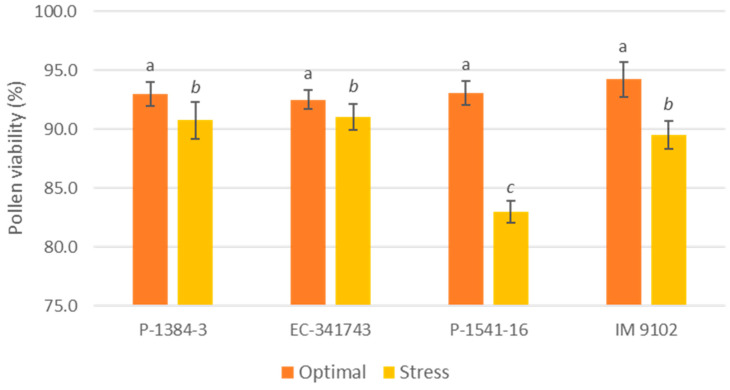
Pollen viability of four field pea genotypes under optimal and heat stress. Columns with different letters indicate significant difference at *p ≤* 0.05 (Fisher LSD test).

A differential response was also noticed for chlorophyll content among tolerant and susceptible genotypes. It is evident that tolerant genotypes were able to maintain their Chl-a, Chl-b, as well as total chlorophyll content at par to control level or showed a non-significant decline under stress environments, whereas a moderate to higher decrement was observed in susceptible genotypes (Figure 1, Figure 2 and Figure 3). A decline in a range of 19–24% could be observed in susceptible genotypes for chlorophyll content due to heat stress. A similar pattern was observed for carotenoid content as well. The tolerant genotypes exhibited a non-significant reduction in the range of 11–13% whereas susceptible genotypes suffered a loss of 26–29% of carotenoid content under heat stress demonstrating higher injury due to stress conditions (Figure 4). The differential behavior in pollen viability was also noticed. Heat stress exposure caused a reduction in pollen viability in P-1384-3 and IM 9102 in the range of 5–11% in heat stress as compared to control temperature, whereas no noticeable change was observed in tolerant genotypes (Figure 5).

### 2.4. Effects of Heat Stress on Biochemical Activities

**Figure 6:**
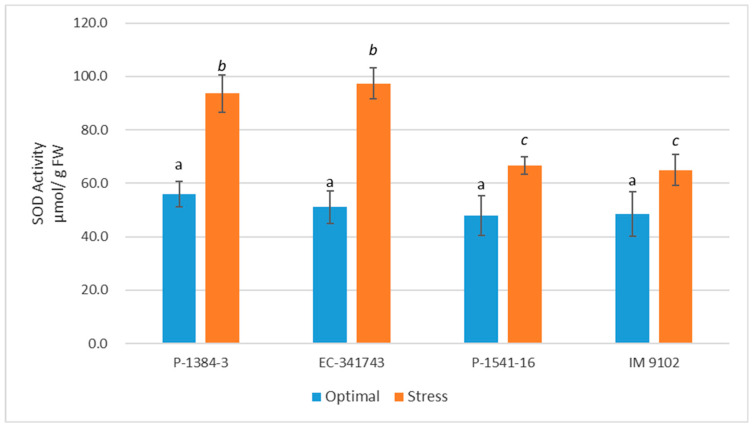
Activity of superoxide dismutase enzyme of four field pea genotypes optimal control and heat stress. Columns with different letters indicate significant difference at *p ≤* 0.05 (Fisher LSD test).

**Figure 7:**
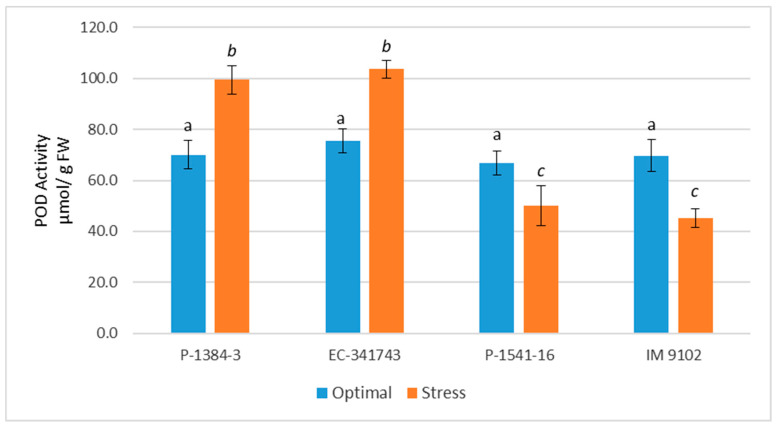
Peroxidase enzyme activity of four field pea genotypes under optimal and heat stress. Columns with different letters indicate significant difference at *p ≤* 0.05 (Fisher LSD test).

**Figure 8:**
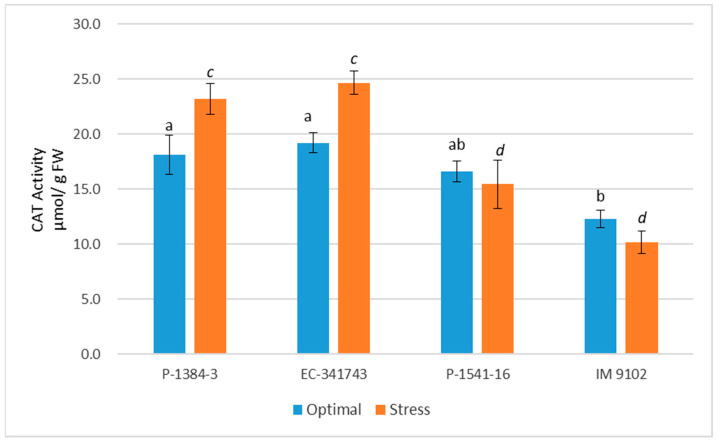
Catalase enzyme activity of four field pea genotypes under optimal and heat stress. Columns with different letters indicate significant difference at *p ≤* 0.05 (Fisher LSD test).

**Figure 9:**
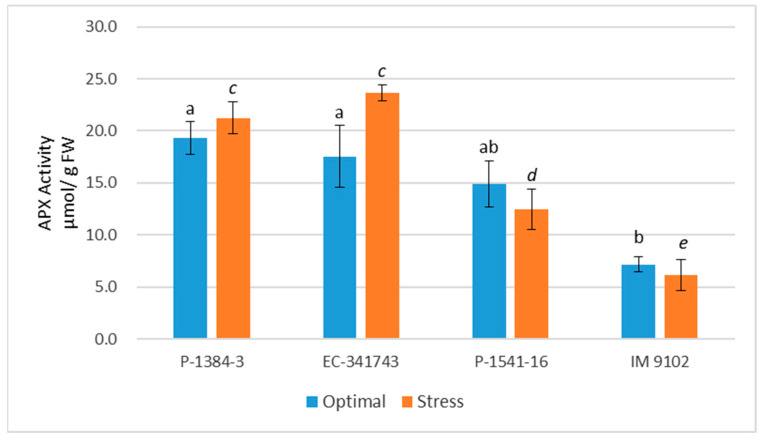
Ascorbate peroxidase enzyme activity of four field pea genotypes under optimal and heat stress. Columns with different letters indicate significant difference at *p ≤* 0.05 (Fisher LSD test).

Both stressed and non-stressed cells produce ROS. It is imperative that ROS be scavenged in order to regulate ROS levels and safeguard cells from damage under stress. There is a highly effective defensive system for scavenging ROS that consists of enzymatic as well as non-enzymatic components. In current study, it is apparent that tolerant genotypes exhibited significant induction in antioxidant enzyme activities under stress conditions to cope with heat stress-induced ROS injury (Figure 6, Figure 7, Figure 8 and Figure 9). Under stress, SOD activity increased in both the tolerant and susceptible genotypes, although the tolerant genotype’s increase was more notable (67% in P-1384-3 while 91% in EC-341743) as compared to susceptible genotypes (39% in P-1541-16 and 34% in IM 9102) (Figure 6). Heat stress stimulated the POD activity by 42% and 37% in P-1384-3 and EC-341743, while a decrease of 25% and 35% was observed in P-1541-16 and IM 9102, respectively (Figure 7). CAT activity was also determined to be slightly increased in tolerant genotypes (28% increase in P-1384-3 as well as EC-341743) under heat stress when compared with control samples (Figure 8), whereas no change in CAT activity was noticed in both susceptible genotypes. Heat stress-induced APX activity by 35% increase in EC-341743 while no change in activity was observed for P-1384-3 (Figure 9). On the other hand, a slight decrease of 17% and 14% was recorded in susceptible genotypes with respect to their APX activity in optimal conditions.

**Figure 10:**
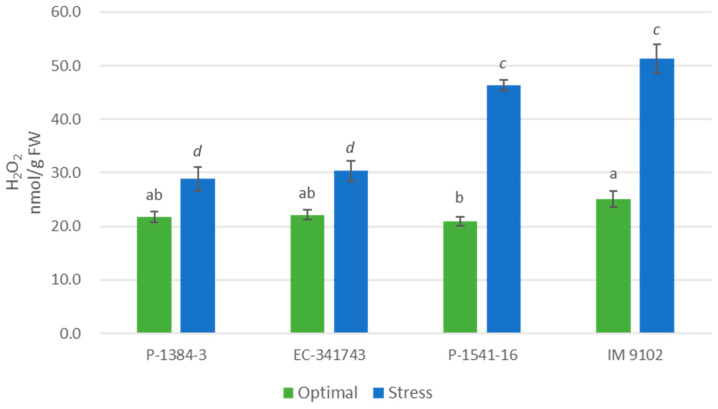
Hydrogen peroxide of four field pea genotypes under optimal and heat stress. Columns with different letters indicate significant difference at *p ≤* 0.05 (Fisher LSD test).

**Figure 11:**
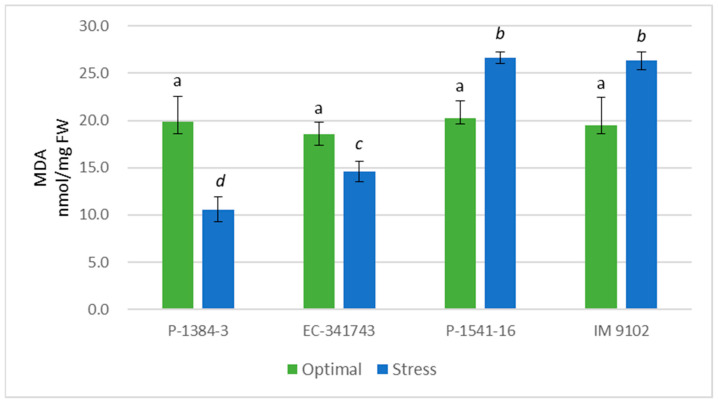
Malondialdehyde content of four field pea genotypes under optimal and heat stress. Columns with different letters indicate significant difference at *p ≤* 0.05 (Fisher LSD test).

Hydrogen peroxide content increased in tolerant as well susceptible genotypes under the influence of heat stress; however, it was observed that where tolerant genotypes showed a marginal increase in H_2_O_2_ content (32% in P-1384-3 and 37% in EC-341743) owing to their induced antioxidant response, a strong rise was noticed for the susceptible genotypes (121% in P-1541-16 and 105% in IM 9102) showing higher oxidative stress injury in these genotypes (Figure 10). Response of MDA content also supported the results obtained above. It was evident that MDA content increased in susceptible genotypes in stress conditions with 31% and 35% higher MDA content in P-1541-16 and IM 9102, respectively, corresponding to controls (Figure 11). At the same time, tolerant genotypes exhibited a decrease of 47% (P-1384-3) and 21% (EC-341743) under stressed conditions with respect to MDA content in optimal conditions.

**Figure 12:**
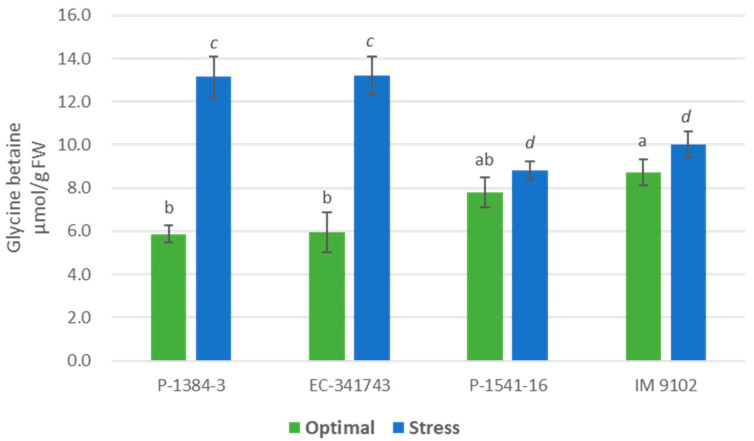
Glycine betaine content of four field pea genotypes under optimal and heat stress. Columns with different letters indicate significant difference at *p ≤* 0.05 (Fisher LSD test).

**Figure 13:**
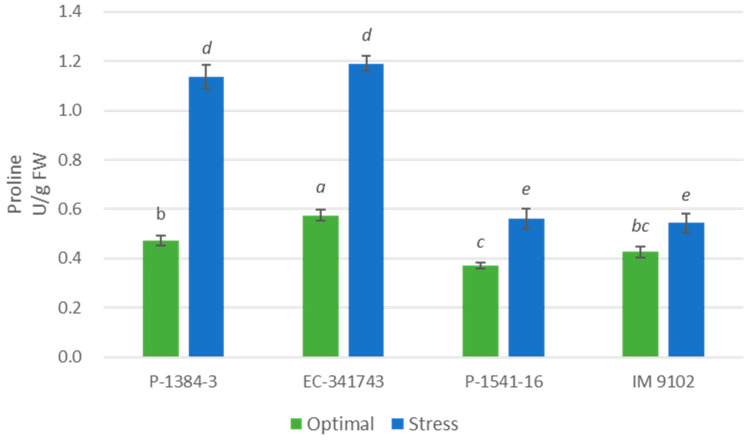
Proline content of four field pea genotypes under optimal and heat stress. Columns with different letters indicate significant difference at *p ≤* 0.05 (Fisher LSD test).

Significant variation with respect to glycine betaine and proline content was observed between two genotypes under optimal and stressed conditions (Figure 12 and Figure 13). A significant increase in osmolytes, i.e., glycine betaine and proline content was noticed in tolerant genotypes under stress conditions. Heat stress caused a marked increase of 124% for P-1384-3 and 121% for EC-341743 in glycine betaine content (Figure 12), while it caused an increase of 140% (P-1384-3) and 107% (EC-341743) in proline content (Figure 13). On the other hand, no change in both the osmolytes could be observed in tolerant genotypes except for P-1541-16, in which a moderate rise of 51% was noticed in proline content.

### 2.5. Principal Component Analysis

**Figure 14:**
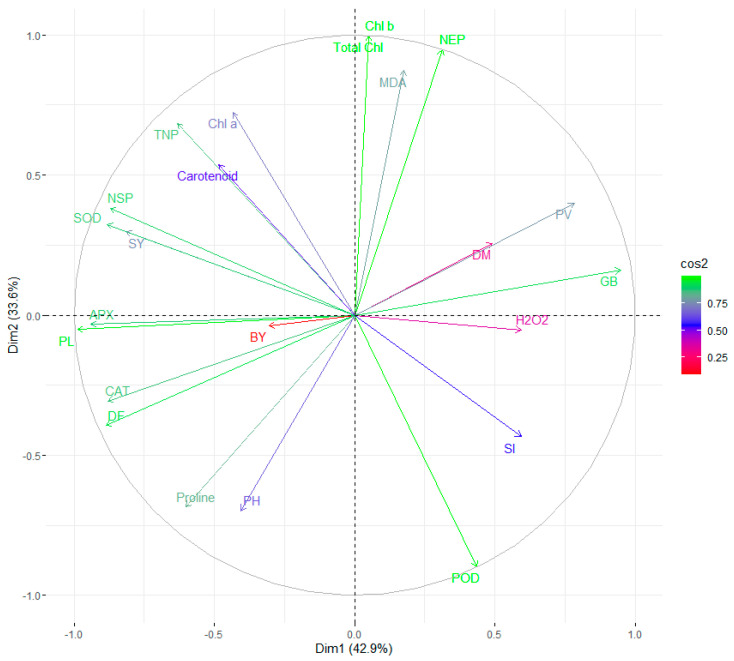
Biplot of different characters of field pea using PC1 and PC2 under non-stress condition.

**Figure 15:**
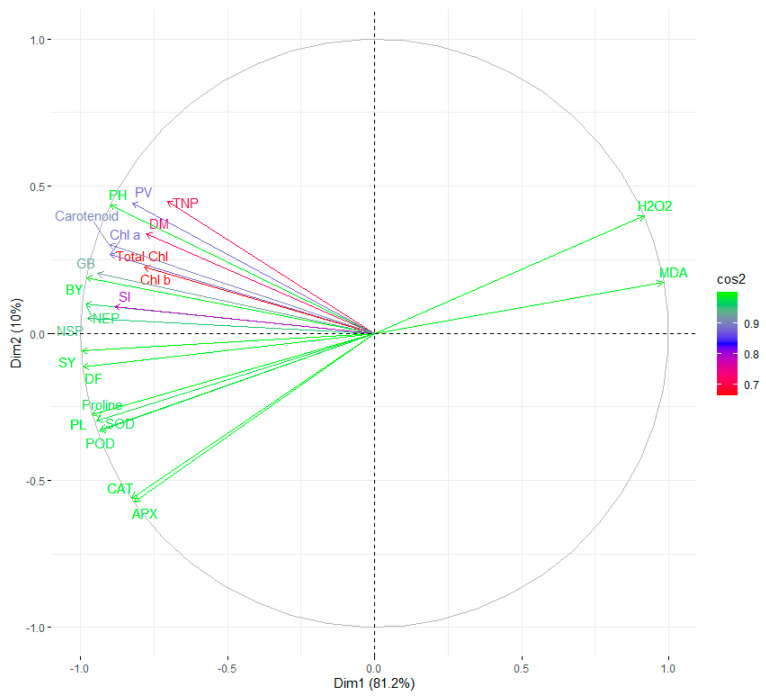
Biplot of different characters of field pea using PC1 and PC2 under heat stress condition.

The principal component analysis (PCA) was performed based on different morphological, physiological, and biochemical traits of field pea in optimal and stress environments which grouped different investigated traits into four principal components (PCs) that account for the entire variance (100%). In optimal conditions, the first two principal components, i.e., PC1 and PC2 showed 76.5% of the total variance, whereas 91.2% of the total variance is explained by the first two PCs in heat stress environment. The first PC explains 42.9% and second PC explains 33.6% of the total variance in optimal condition, while in stress condition, 81.2% and 10% variance is explained by the first PC and second PC, respectively. Biplots of investigated traits of field pea in optimal and stress environments are depicted in Figure 14 and Figure 15, respectively. The biplot for optimal conditions represents a strong correlation of SY with SOD and NSP, while under heat stress conditions, SY showed a strong correlation with DF, NEP, BY, proline, PL, NSP, SOD and POD, as there was a very small angle between corresponding vectors of above traits. The SY also had a significant negative association with MDA in heat stress conditions, as they possess the largest angle between the vectors of SY and MDA.

## 3. Discussion

The field pea is known as vulnerable to climatic fluctuations, especially to the high-temperature stress. In tropical regions, the crop frequently faces terminal heat stress demonstrating the necessity for the creation of heat-tolerant cultivars [29]. Increased temperatures that are higher than what plants need to thrive hamper growth and yield by drastically reducing photosynthesis [9,30]. According to global climate models, the mean ambient temperature will be raised by 1.5 °C during the next 20 years [31]. While prioritizing high yielding capacity in favorable environments, the majority of crop breeding efforts have instead relied on a small number of progenitor germplasm, which has resulted in a reduction in the genetic diversity of stress resistance traits such as heat stress tolerance. The impacts of heat stress on reducing photosynthesis, affecting respiration, inactivating enzymes, and rupturing membranes can significantly reduce biomass production [32,33]. However, the heat stress-induced severity depends on duration and intensity of stress on genotypes [34]. Practical ramifications of a better understanding of morpho-physiological and biochemical characteristics linked with heat stress tolerance include identification of different tolerance mechanism and their application in alleviation treatments [35,36]. To maintain crop production under the current and future changing climates, thermo-tolerance requires a multidisciplinary, holistic strategy that integrates the results of biochemical, physiological, breeding, and agronomic interventions [37]. The excess production of ROS that leads to lipid peroxidation, DNA damage, protein oxidation, and cell death is seen in plants during heat stress [38,39]. Generation of ROS has already been reported to play a key role in activation of a mechanism eventually leading to plant adaptation to several abiotic stresses [40]. One of such responses includes activation of antioxidant enzymes. However, several other factors also play a pivotal role in plant survival under heat stress including osmolytes, which are important for maintaining osmotic balance of a cell as well as cell redox state by acting as antioxidants, pigments such as chlorophyll, and carotenoids which maintain photosynthetic efficiency of plants under stress as well act as a precursor for antioxidants [41]. In the current study, we have studied the effect of heat stress on metabolic responses of tolerant and susceptible field pea genotypes in order to gain insights into the mechanism of tolerance in differential genotypes.

The genotypes exposed to heat stress showed reduction in almost all the growth and yield component traits, in general. For SY, a considerable decrease in performance was detected in susceptible genotypes (P-1541-16 by 69% and IM 9102 by 53%) by exposure to heat stress. Tolerant genotypes (EC 341743 and P-1384-3) showed minimum reduction in SY under heat stress as compared to susceptible ones. Among all the genotypes, similar patterns were also recorded for the NEP, SI, and BY under heat stress. This indicated that the performance of seed yield is supported by the seed number per pod, seed index, and biomass of plants. Similar findings for one or more characters were reported by Hall [42]; Sadras et al. [43]; Atung [44]. Heat stress also stimulates a lower reduction in PL and NSP in tolerant genotypes (EC 341743 and P-1384-3) compared with susceptible genotypes. Tafesse et al. [45] have shown that heat stress reduced plant height, pod number, pod set rate, and seed yield of pea genotypes. They also observed differential responses of pea varieties to different growth and fruiting characteristics under heat stress. Jiang et al. [46] also reported a reduction in the seed yield of pea genotypes due to acceleration in plant life cycle and decrease in pod number and seed size under heat stress.

In order to understand the photosynthetic efficiency of contrasting genotypes under stressed conditions, the chlorophyll and carotenoid content has also been studied. In order to select the photo-synthetically effective genotypes under heat stress, features such as high chlorophyll content, photosystem-II function, and stomatal conductance are crucial [11]. It was determined that tolerant genotypes maintained their pigments level at par with control while a decrease in content was noticed for susceptible genotypes which may have in turn affected their photosynthesis ability under stress conditions. The primary photosynthetic pigment, chlorophyll, helps to absorb light energy and facilitates photosynthesis [47]. The senescence of the leaves is brought on by heat stress causing a reduction in chlorophyll content [48]. Increased chlorophyll retention during heat stress may be considered as a measure of heat stress resistance. Consequently, selecting heat-tolerant genotypes could benefit from genetic diversity for chlorophyll content. Another essential character for choosing genotypes for heat tolerance is membrane stability, which is determined by electrolyte/ion leakage [49]. Similar observations have been obtained by Chaudhary et al. [50] also while studying impact of heat stress on tolerant and susceptible urdbean genotypes. Similar to this, Devi et al. [51] observed that in high-temperature conditions of outdoor and growth chambers, heat-tolerant chickpea genotypes displayed greater chlorophyll content than heat-sensitive genotypes. Similar findings have also been reported previously in lentil plants subjected to heat stress [52,53].

The above results also were in line with the high percentage of pollen viability (97–98%) in tolerant genotypes. Karwa et al. [54] also reported a high percentage of pollen viability in tolerant rice genotypes under heat stress, whereas susceptible genotypes reflected a significant decrease of 40–51%. Our results are also in agreement with another study conducted on wheat seedlings in which the authors reported higher pollen viability under heat stress situations in heat-tolerant wheat line M3 based on 2,3,5-triphenyl tetrazolium chloride reduction test as compared to susceptible wheat line M9 [55]. One of the key indicators of effective reproductive function includes pollen viability levels, which predict high pod and seed setting and an increase in yield under heat stress [56].

As evident from the results, tolerant genotypes exhibited activation of their antioxidant defense system by enhancing the activities of SOD, POD, and CAT, while in susceptible genotypes, only SOD expression was determined to be increased. By increasing antioxidant activity in response to heat and drought stress, ROS scavenging is frequently induced, and this is associated with stress tolerance [57]. As superoxide is dismutated into H_2_O_2_ and O_2_ by the important ROS-scavenging enzyme SOD, a rise in SOD activity has been linked with an increase in protection against the harm brought on by oxidative stress [58]. Similar to this, the stimulation of CAT activity under heat stress in thermo-tolerant genotypes demonstrates their innate ability to express CAT when necessary. This type of conduct was not displayed by any of the vulnerable genotypes. Higher plants have CAT localized in the peroxisomes, which is responsible for breaking down H_2_O_2_, which is also formed outside of the chloroplasts by oxidases in the peroxisomes that synthesize H_2_O_2_. Despite its limited localization, it might be crucial in eliminating H_2_O_2_. Hence, an increase in CAT activity is directly linked to an increase in stress tolerance, since it is associated with scavenging H_2_O_2_ [59]. Likewise, POX is among the enzymes that scavenge H_2_O_2_ generated by the dismutation of O_2_^·^ catalyzed by SOD, hence its upregulation plays a significant role in ROS detoxification. In addition to scavenging H_2_O_2_ generated in chloroplasts, peroxidases also play a role in [60]. The elevated activity of three crucial antioxidant enzymes in tolerant field pea genotypes provides a rational explanation for the reason behind these genotypes being able to scavenge superoxide anions produced in plants as a result of heat stress more effectively. One of the mechanisms for greater antioxidant defense and ultimately improved tolerance to abiotic stimuli has been shown to be the overexpression of the SOD and APX genes, which are used in scavenging ROS [61]. Our findings concur with recent studies that determined SOD, POX, and CAT to be the major detoxifying enzymes that work in concert with APX and GR of the ascorbate–glutathione cycle to scavenge ROS during abiotic stresses [62,63,64,65]. In a recent report, Iqbal et al. [41] have also asserted that the decrease in oxidative stress in wheat plants studied under heat stress was due to enhanced action of antioxidative enzymes SOD, APX, CAT, GR and GR gene expression. According to Basu et al. [66], heat-sensitive genotypes of black gram and chickpea were driven to express higher levels of SOD and POX as a defense against ROS. In contrast, heat-tolerant genotypes are naturally resilient to stress and consequently produce less ROS.

The results obtained above are further substantiated and supported by the results of oxidative stress indicators, H_2_O_2_ and MDA levels. Under heat stress, tolerant genotypes displayed regulated H_2_O_2_ levels, indicating less severe oxidative damage. Almost unchanged H_2_O_2_ and MDA content in tolerant genotypes under stress conditions might be due to higher SOD, CAT, and POD activity in its tissues. As opposed to susceptible genotypes where only POD activity is induced, it may be hypothesized that in P-1384-3 and EC-341743, H_2_O_2_ detoxification was efficiently governed by simultaneous action of SOD, CAT, and POD. This may be the explanation for the sensitive behavior of P-1541-16 and IM 9102 in heat-stressed environments. MDA concentration is frequently utilized as a marker of lipid peroxidation brought on by diverse abiotic stresses in plant tissues. The ability of tolerant plants to withstand environmental challenges such as heat seems to be reflected in the unchanged lipid peroxidation. Other researchers also observed similar findings linking lipid peroxidation with antioxidative system function [67,68,69]. Ding et al. [70] also reported a direct relationship between the decreases in H_2_O_2_ content with an increase in antioxidant enzymes under heat stress in *Phragmites communis*. Chaudhary et al. [50] also reported low MDA content in heat-tolerant urdbean genotypes tested under field and growth chamber conditions. Our findings are also in concurrence with Devi et al. [51], who reported the same results in chickpea cultivars under heat stress.

Under conditions of drought, salt, flooding, and cold stress, many plants acquire a variety of suitable osmolytes, such as proline and different sugars, and these osmolytes serve as osmoprotectants for plant stress tolerance [71,72]. In the present study, tolerant genotypes demonstrated accumulation of proline and glycine betaine in their tissues under stress conditions while content remained unchanged in susceptible genotypes. The protective function of proline and glycine betaine under stress involves direct scavenging of ROS, stabilization of proteins as well as antioxidant enzymes, balancing intra-cellular redox homeostasis (e.g., ratio of NADP^+^/NADPH and GSH/GSSG), and promotion of cell-signaling and protection of photosynthetic pigments [73,74]. Therefore, the higher proline content in tolerant genotypes may improve plant survival under stress environments and may also serve as a biochemical marker for early genotype screening. Shahid et al. [75] reported a significant role of proline in imparting salinity tolerance in two pea (*Pisum sativum* L.) cultivars (cv. L-888 and cv. Round). Similarly, the function of glycine betaine in heat tolerance is advocated by various studies [76,77,78]. An increase in GB and proline have been reported to impart heat stress in chickpea and *S. lycopersicum* seedlings [79,80].

The PCA revealed considerable variability among the traits with two major PCs around 76.5% and 91.2% of total variation in optimal and stress conditions, respectively. These results revealed that the traits that contribute significantly to the first two PCs are principal discriminatory traits. The biplot analysis revealed information about the association of investigated traits. These findings are in accordance with Parihar et al. [81], Pratap et al. [82], Mohapatra et al. [83]. As evident from the result, significant positive associations were detected between SY and some related characters. SY was determined to have a strong positive correlation with NEP, BY, Proline, PL, NSP, SOD and POD under heat stress, suggesting that positive selection on NEP, BY, Proline, PL, NSP, SOD, and POD may indirectly assist in the selection of suitable best performing genotype under heat stress, while in the optimalconditions, SOD and NSP contributed to indirect selection for choosing the best-performing genotype. In addition, the occurrence of a significant negative correlation between SY and MDA under heat stress indicates that a reduction in MDA may increase SY in field pea [83,84,85].

## 4. Materials and Methods

### 4.1. Plant Materials

A previous study with 140 field pea germplasm collected from CSAUAT, Kanpur; IIPR, Kanpur and CCSHAU, Hisar was conducted at Banda University of Agriculture and Technology, Banda and two HS and two HT genotypes under high temperature were selected for further study [86]. These genotypes include one cultivar (IM 9102), two breeding lines (P-1384-3 and P-1541-16), and one exotic collection (EC 341743). Among them, genotypes P-1384-3, EC 341743 are heat tolerant, and P-1541-16, IM 9102 are heat susceptible.

### 4.2. Experimental Design and Treatments

**Figure 16:**
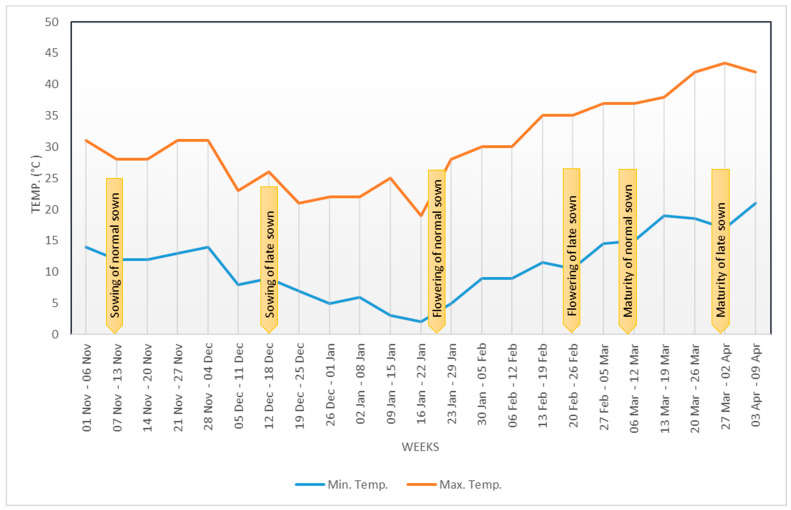
Weekly temperature data recorded during cropping period at the experimental site.

The experiment was conducted at Research Farm, College of Agriculture, Banda University of Agriculture and Technology, Banda during the winter season (November 2021 to April 2022). The experimental site is located between latitudes 24°53′–25°55′ N and longitudes 80°07′–81°34′ E. Climatic conditions of experimental site are categorized as semi-arid with hot summers and cold winters. The soil composition of experimental site was clay loam with pH 8.40, low content of organic matter (0.33%), nitrogen, and phosphorus. The meteorological data for the growing season are shown in Figure 16. The experiment was laid out to evaluate four pea genotypes in Randomized Block Design with three replicates. The experiments were sown in two sets, viz., timely sown, i.e., in optimal condition (12 November 2021) and late sown, i.e., under heat stress condition (12 December 2021). Each genotype sown in six rows with a row length of 4 m and spacing of 30 cm from row to row as well as of 15 cm from plant to plant. Recommended cultivation practices were followed to obtain a healthy crop.

### 4.3. Data Collection

#### 4.3.1. Plant Growth and Yield Component Traits

From each genotype in optimal and stress environment, 5 randomly sampled competitive plants were selected for noting down observations on ten quantitative traits, except days to 50% flowering and days to maturity. These two traits were recorded on plot basis. The observations for the following traits, viz., days to 50% flowering and maturity, height of plant (cm), the total number of pods per plant, effective pod number per plant, pod length (cm), seed number per pod, seed index (g), biological yield per plant (g) and seed yield per plant (g) were recorded at different growth stages.

#### 4.3.2. Physiological Traits

Entire physiological traits were determined at pod-filling stage of field pea on non-stressed as well as stressed plants. Several physiological traits, namely photosynthetic pigments (chlorophyll and carotenoids), and pollen viability (%) were studied.

##### Photosynthetic Pigments

For quantitative analysis of chlorophyll and carotenoid in field pea, the 100 mg leaf sample was homogenized in 80% acetone and centrifuged at 8000 rpm for 5 min at room temperature. Absorbance was measured in a spectrophotometer at 663 nm, 645 nm, and 470 nm from 2 mL of supernatant. Chlorophyll content was estimated as per formula from Arnon [87] and expressed as mg/g FW. Carotenoid content was estimated by the formula of Lichtenthaler [88] and expressed in μg/100 mL.

(1) $$\mathrm{Chl}\;‘a’=12.7\;\times \;A_{663}-2.69\;\times \;A_{645}\;\times \;\frac{V}{1000\times W},$$

(2) $$\mathrm{Chl}\;‘b’=22.9\;\times \;A_{645}-4.68\;\times \;A_{663}\;\times \;\frac{V}{1000\times W},$$

(3) $$\mathrm{Total}\;\mathrm{Chlorophyll}=(20.2\;\times \;A_{645})+(8.02\;\times \;A_{663})\;\times \;\frac{V}{1000\times W},$$

(4) $$\mathrm{Carotenoid}=\frac{1000\times \;A470\;–\;\left(1.82\times \;\mathrm{Chl}\;a\right)–\;\left(85.02\times \;\mathrm{Chl}\;b\right)}{198},$$

where

A = Absorbance;

*V* = Final volume of 80% acetone (mL);

*W* = Weight of sample (g).

##### Pollen Viability

To assess PV, pollen grains excised from anthers and stained on the glass slide with 1% acetocarmine. Pollen grains were counted from each slide and the percentage was calculated based on the ratio of viable (stained) and non-viable (unstained) pollen grains.

#### 4.3.3. Biochemical Analyses

Various parameters such as proline content, glycine betaine, malondialdehyde, hydrogen peroxide and antioxidant enzyme activities such as SOD, POD, CAT and APX were analyzed.

##### Proline Content

Proline content estimated as per protocol proposed by Bates et al. [89]. Briefly, 200 mg of fresh leaves were homogenized in 3% sulfosalicylic acid (pH 7.5) and centrifuged at 12,000 rpm for 15 min at 4 °C. The 400 μL clear supernatant was mixed with acid ninhydrin, 400 μL glacial acetic acid and 400 μL phosphoric acid, and then incubated at 100 °C for 1 h. Then, 800 μL of toluene was added to mixture and absorbance was recorded at 520 nm.

##### Glycine Betaine

Fresh leaf samples were placed in oven at 85 °C for 6–8 h. A total of 200 mg of the dried sample was weighed and crushed it with 10 mL of distilled water. This mixture was kept in the shaker and let stand overnight. It was centrifuged at 8000× *g* for 5 min at room temperature. The supernatant and 2 N sulphuric acid in the ratio of 1:1 were taken and the absorbance was measured at 365 nm.

##### Malondialdehyde

MDA activity was estimated with Heath and Packer [90] method with minor modification. The 200 mg leaf sample was homogenized in 1 mL of 5% TCA (Trichloroacetic acid) and centrifuged at 12,000 RPM for 20 min at a temperature of 4 °C. To analyze MDA content, 1 mL of supernatant was transferred to test tubes and 4 mL of 0.5% TBA (Thiobarbituric acid) was added in 20% TCA, and this assay was incubated in a water bath at 96 °C for 30 min. Immediately after incubation, these tubes were placed on ice for 10 min to stop the reaction. Then, the tubes were centrifuged at 2000× *g* RPM for 10 min at a temperature of 4 °C. Absorbance was measured at 600 nm and 532 nm using UV–vis spectrophotometer.

##### Hydrogen Peroxide

It was measured with a method proposed by Sinha [91] with some modifications. To measure hydrogen peroxide, 200 mg of fresh leaves were weighed and ground with 2 mL of phosphate buffer with a pH 7. The contents were placed into 2 mL tubes and centrifuged at 12,000 RPM at room temperature for 15 min. Clear supernatant was used to measure the H_2_O_2_ activity. To estimate H_2_O_2_, 1 mL of sample and 1 mL of 10 mM phosphate buffer (pH 7.0) were taken. To start the reaction, 2 mL of 5% K_2_Cr_2_O_7_ with glacial acetic acid in the reaction were mixed. The absorbance was measured with a UV–vis spectrophotometer at 570 nm.

##### Antioxidant Enzyme Activities

- Superoxide Dismutase Activity

SOD activity was determined with a method designed by Marklund and Marklund [92] with some minor modifications. The 200 mg of fresh leaves were crushed with a mortar and pestle with 2 mL of sodium phosphate extraction buffer (100 mM) at pH 7.5. The content centrifuged at 12,000 RPM for 15 min at 4 °C temperature and the clear supernatant was separated. The activity was measured by taking 1.5 mL of Tris HCL + 500 μL EDTA and adding 200 μL of supernatant. Lastly, to initiate the activity, 1 mL of pyrogallol was mixed into the reaction assay. Absorbance was recorded at 420 nm using the UV–vis spectrophotometer after every 30 s up to 3 min.

- Peroxidase Activity

POD activity was measured with a method suggested by Shannon et al. [93] with some minor modifications. Extraction was conducted in the same way as for SOD, and then the supernatant was separated. For activity analysis, 3 mL of guaiacol was added to the cuvette, and 200 μL of supernatant was also added. To start the reaction, 100 μL H_2_O_2_ was added at the end. The absorbance was measured at 470 nm using UV–vis spectrophotometer after every 30 s up to 3 min.

- Catalase Activity

CAT activity was determined with a method of Chance and Maehly [94] with some modifications. For estimation of CAT activity, 200 mg fresh leaves homogenized in 2 mL of 50 mM phosphate buffer containing 1% polyvinylpyrrolidone, pH 7.5. CAT activity was measured by taking 1.8 mL extraction buffer + 1 mL supernatant and 60 μL H_2_O_2_. The absorbance of the reaction solution at 240 nm was recorded every 30 s.

- Ascorbate Peroxidase Activity

To estimate APX activity, fresh leaves were crushed in a medium consisting of 50 mM phosphate buffer (pH 7.0). APX activity was determined by adopting the method of Dixit et al. [95] with some modifications. For the measurement of APX, the assay solution contained an assay buffer consisting of 0.5 mM ascorbic acid, 1% PVP, and 50 mM phosphate buffer, H_2_O_2_ (60 μL) and 200 μL supernatant. Absorbance was measured after every 30 s at 290 nm [96].

### 4.4. Statistical Analysis

The replicate-wise means of each genotype for different traits were used for the analysis of the observed data. All results were expressed as mean ± SE (standard error) in tables and graphs with three replicates. All the graphs were drawn using the program MS Excel version 2016. The two-factor analysis of variance and principal component analysis estimated using the statistical program R (version 4.1.3). The significant differences between means at *p* ≤ 0.05 were determined using Fisher least significant difference (LSD) test.

## 5. Conclusions

The significant effect of heat stress was recorded in the genotypes for the studied traits. The performance of plant growth, yield component, physiological, and biochemical traits were considerably reduced under heat stress over the optimal conditions. PCA revealed that considerable variability contributed by the first two major PCs around 76.5% and 91.2% of total variation in optimal and stress conditions, respectively. Significant reduction was detected for SY in susceptible genotypes (P-1541-16 and IM 9102) in heat stress, whereas tolerant genotypes (EC 341743 and P-1384-3) showed less reduction. Similar patterns of reduction in the NEP, SI, and BY in heat stress were also observed for susceptible genotypes as compared to tolerant ones. On the other hand, significant reductions were also observed for photosynthetic pigments (chlorophyll and carotenoid) in heat stress. A remarkable enhancement occurs in the activities of antioxidants and osmoprotectants in tolerant genotypes compared to sensitive ones. The SY was determined to have significant and positive association with SOD and NSP under optimal conditions, whereas under heat stress conditions, it was positively associated with NEP, BY, proline, seed number per pod, SOD, and POD. Based on the results, the higher concentration of osmoprotectants with higher anti-oxidant enzymes activities and lower ROS accumulation was the key factor for considering heat-tolerant genotypes of field pea, and these results were helpful in understanding the mechanisms of adaptability of plants to heat stress.

## Figures and Tables

**Table 1:** Mean sum of squares for morphological, physiological, and biochemical traits of four field pea genotypes.

S. No.	Characters	Abbreviations	Sources of Variation		
			G	T	G × T
			*df* = 3	*df* = 1	*df* = 3
1	Days to 50% flowering	DF	31.90 **	105.06 **	0.73 ^NS^
2	Days to maturity	DM	39.17 **	756.26 **	19.75 *
3	Plant height (cm)	PH	1760.90 **	8340.30 **	411.50 **
4	Total number of pods per plant	TNP	16.99 ^NS^	743.93 **	36.15 *
5	Number of effective pods per plant	NEP	11.70 ^NS^	750.76 **	17.47 *
6	Pod length (cm)	PL	1.80 **	3.96 **	0.19 ^NS^
7	Number of seeds per pod	NSP	3.32 **	2.48 **	0.07 ^NS^
8	Seed index (g)	SI	2.00 *	23.52 **	4.41 **
9	Biological yield per plant (g)	BY	303.3 **	3366.90 **	56.00 *
10	Seed yield per plant (g)	SY	85.12 **	130.53 **	25.98 *
11	Chlorophyll a (mg/g FW)	Chl-a	61.71 **	48.91 **	4.03 ^NS^
12	Chlorophyll b (mg/g FW)	Chl-b	12.83 **	23.56 **	3.81 **
13	Total chlorophyll (mg/g FW)	Total Chl	9.99 **	18.32 **	2.97 **
14	Carotenoid (μg/100 mL)	Carotenoid	8.55 **	9.47 **	0.38 ^NS^
15	Pollen viability (%)	PV	14.46 **	85.79 **	14.96 **
16	Superoxide dismutase (µmol/g FW)	SOD	405.40 **	3541.10 **	210.90 ^NS^
17	Peroxidase (µmol/g FW)	POD	970.95 **	245.00 *	1134.24 **
18	Catalase (µmol/g FW)	CAT	94.78 **	13.20 *	16.14 **
19	Ascorbate peroxidase (µmol/g FW)	APX	173.63 **	5.33 ^NS^	14.23 ^NS^
20	Hydrogen peroxide (nmol/g FW)	H_2_O_2_	302.12 **	2236.13 **	221.79 **
21	Malondialdehyde (nmol/mg FW)	MDA	71.52 **	0.00 ^NS^	62.90 **
22	Glycine betaine (µmol/g FW)	GB	9.57 **	128.64 **	28.34 **
23	Proline (U/g FW)	Proline	0.37 **	1.26 **	0.16 **

* Significant at *p* ≤ 0.05, ** Significant at *p* ≤ 0.01, NS—Non-significant at *p* ≤ 0.05, *df—*degree of freedom, G—Genotypes, T—Treatments (optimal and heat stress), G × T—Genotypes and treatments interaction.

**Table 2:** Mean value of field pea genotypes for plant growth and yield component traits under control and heat stress conditions.

**S.N.**	**Genotypes**		**DF**		**DM**		**PH**		**TNP**	
			**Optimal**	**Stress**	**Optimal**	**Stress**	**Optimal**	**Stress**	**Optimal**	**Stress**
1	P-1384-3		76.5 ± 1.5	72.5 ± 0.5	118.0 ± 3.0	105.5 ± 2.5	116.7 ± 1.1	88.7 ± 1.3	38.0 ± 2.0	22.2 ± 0.3
2	EC-341743		77.5 ± 2.5	72.0 ± 2.0	116.0 ± 2.0	108.0 ± 1.0	151.6 ± 3.8	77.15 ± 2.9	33.0 ± 3.6	24.7 ± 2.1
3	P-1541-16		72.5 ± 0.5	66.5 ± 1.5	115.0 ± 1.0	98.0 ± 1.0	83.25 ± 2.0	47.15 ± 2.9	35.9 ± 0.7	14.7 ± 1.5
4	IM 9102		72.5 ± 0.5	67.5 ± 0.5	122.5 ± 0.5	105.0 ± 2.0	116.9 ± 1.1	72.8 ± 2.6	32.1 ± 2.9	22.9 ± 2.1
**S.N.**	**Genotypes**		**NEP**		**PL**		**NSP**		**SI**	
			**Optimal**	**Stress**	**Optimal**	**Stress**	**Optimal**	**Stress**	**Optimal**	**Stress**
1	P-1384-3		31.1 ± 0.7	20.2 ± 0.4	5.8 ± 0.4	5.2 ± 0.3	5.5 ± 0.5	4.7 ± 0.4	13.5 ± 0.5	14.0 ± 0.5
2	EC-341743		29.4 ± 0.8	19.7 ± 1.9	5.6 ± 0.2	5.0 ± 0.4	4.2 ± 0.4	3.8 ± 0.2	14.6 ± 0.6	11.8 ± 0.8
3	P-1541-16		31.1 ± 1.6	12.3 ± 0.9	5.0 ± 0.2	3.6 ± 0.2	3.6 ± 0.2	2.5 ± 0.3	13.5 ± 0.3	10.7 ± 0.7
4	IM 9102		31.0 ± 2.6	15.6 ± 2.1	4.8 ± 0.2	3.5 ± 0.3	3.7 ± 0.2	2.9 ± 0.4	15.5 ± 0.5	11.0 ± 0.5
**S.N.**		**Genotypes**	**BY**				**SY**			
			**Optimal**		**Stress**		**Optimal**		**Stress**	
1		P-1384-3	66.5 ± 4.1		47.0 ± 2.6		21.3 ± 1.9		19.2 ± 0.8	
2		EC-341743	64.2 ± 2.0		41.4 ± 3.4		18.3 ± 2.8		17.6 ± 0.6	
3		P-1541-16	53.3 ± 3.1		19.3 ± 3.0		15.8 ± 1.8		4.9 ± 0.5	
4		IM 9102	65.8 ± 4.2		31.1 ± 2.9		17.1 ± 3.5		8.0 ± 0.9	

Values are expressed as mean ± SE (standard error).

## Data Availability

Not applicable.
