# Correction: Sharma et al. Morpho-Physiological and Biochemical Responses of Field Pea Genotypes under Terminal Heat Stress. *Plants* 2023, *12*, 256

**DOI:** 10.3390/plants15132060

**Published:** 2026-07-02

**Authors:** Vijay Sharma, Chandra Mohan Singh, Vishal Chugh, Kamaluddin, Pawan Kumar Prajapati, Anuj Mishra, Prashant Kaushik, Parmdeep Singh Dhanda, Alpa Yadav, Satyendra

**Affiliations:** 1 Department of Genetics and Plant Breeding, College of Agriculture, Banda University of Agriculture and Technology, Banda 210001, India; vijay.buat@gmail.com (V.S.); cmsingh.gpb@gmail.com (C.M.S.); kamaluddinpbg@gmail.com (K.); pkprajapatii88@gmail.com (P.K.P.); anujmishra8932@gmail.com (A.M.); bhusatyendra91@gmail.com (S.); 2 Department of Basic and Social Sciences, College of Horticulture, Banda University of Agriculture and Technology, Banda 210001, India; 3 Independent Researcher, 46022 Valencia, Spain; 4 Department of Biochemistry, Punjab Agricultural University, Ferozepur Road, Ludhiana 141027, India; parmdeepdhanda@gmail.com; 5 Department of Chemistry, Ranchi University, Ranchi 834001, India; alpa.yadav21@gmail.com

In the publication [1], there was an error regarding the affiliation for Prashant Kaushik. The updated affiliation should be as follows: Independent Researcher, 46022 Valencia, Spain.

The authors state that the scientific conclusions are unaffected. This correction was approved by the Academic Editor. The original publication has also been updated.
